# Cluster randomized trial comparing school-based mass drug administration schedules in areas of western Kenya with moderate initial prevalence of *Schistosoma mansoni* infections

**DOI:** 10.1371/journal.pntd.0006033

**Published:** 2017-10-23

**Authors:** Diana M. S. Karanja, Emmy K. Awino, Ryan E. Wiegand, Edward Okoth, Bernard O. Abudho, Pauline N. M. Mwinzi, Susan P. Montgomery, W. Evan Secor

**Affiliations:** 1 Neglected Tropical Diseases Branch, Centre for Global Health Research, Kenya Medical Research Institute, Kisumu, Kenya; 2 Division of Parasitic Diseases and Malaria, Centers for Disease Control and Prevention, Atlanta, GA, United States of America; Swiss Tropical and Public Health Institute, SWITZERLAND

## Abstract

**Background:**

Mass drug administration (MDA) using praziquantel is the WHO-recommended approach for control of schistosomiasis. However, few studies have compared the impact of different schedules of MDA on the resultant infection levels. We wished to evaluate whether annual MDA was more effective than less frequent treatments for reducing community-level prevalence and intensity of *Schistosoma mansoni* infections.

**Methods:**

We performed a cluster randomized trial (ISRCTN 14849830) of 3 different MDA frequencies over a 5 year period in 75 villages with moderate (10%-24%) initial prevalence of *S*. *mansoni* in school children in western Kenya. Praziquantel was distributed by school teachers to students either annually, the first 2 years, or every other year over a 4 year period. Prevalence and intensity of infection were measured by stool examination in 9–12 year old students using the Kato-Katz method at baseline, each treatment year, and for the final evaluation at year 5. *S*. *mansoni* prevalence and intensity were also measured in first year students at baseline and year 5.

**Results:**

Twenty-five schools were randomly assigned to each arm. *S*. *mansoni* prevalence and infection intensity in 9–12 year old students significantly decreased within each arm from baseline to year 5 but there were no differences between arms. There were no differences in infection levels in first year students either within or between arms.

**Conclusions:**

Strategies employing 2 or 4 rounds of MDA had a similar impact in schools with moderate initial prevalence, suggesting that schistosomiasis control can be sustained by school-based MDA, even if provided only every other year.

## Author summary

The World Health Organization (WHO) guidelines recommend mass drug administration (MDA) of praziquantel for control of schistosomiasis. The target group for and the frequency of treatment are based on infections levels in school age children at the initiation of the control program. However, these guidelines have not been rigorously evaluated. Through the Schistosomiasis Consortium for Operational Research and Evaluation (SCORE), we had the opportunity to compare the impact of school-based treatment in an area of western Kenya with moderate (10%-24%) initial prevalence of *Schistosoma mansoni* infections. We found that providing MDA every year or every other year over a 4 year period provided similar benefits for reducing prevalence and intensity of infection in the final evaluation at year 5. Annual or biennial MDA moved villages from the “moderate risk” to the “low risk category” prescribed in the WHO guidelines. The results from this study and parallel SCORE studies in other African countries should provide the evidence base necessary for improved WHO guidelines for schistosomiasis.

## Introduction

Passage of World Health Assembly (WHA) resolution 54.19 helped stimulate increased efforts, resources, and research to control morbidity caused by schistosomiasis. The main approach for schistosomiasis control recommended by the World Health Organization (WHO) is mass drug administration (MDA) using praziquantel [1, 2]. Praziquantel has been used successfully over the past 30 years in many countries and is appropriate for MDA as it is inexpensive, well-tolerated, and safe in persons who are not infected [3].

The current WHO guidelines for schistosomiasis MDA suggest different strategies based on prevalence of infection in school-age children (SAC) [1, 2]. Communities with < 10% egg positive individuals among SAC are considered at low risk for morbidity caused by schistosomiasis; the recommended action is two treatments during primary school. Communities with SAC egg positive prevalence between 10% and 50% are considered moderate risk and every other year MDA is recommended. High risk communities are those with > 50% egg positive SAC and annual MDA is recommended. Both SAC in and out of school are targeted and treatment is also recommended for adults with high likelihood of infection in moderate and high risk communities.

These guidelines were developed with the primary goal of preventing severe morbidity associated with high-intensity infections and at a time when the supply of praziquantel was much more restricted than it is currently. In recent years, there has been a growing recognition of an association of morbidity even with low intensity infections and a more than six-fold increase in donation of praziquantel tablets by pharmaceutical companies and purchases by bilateral organizations [4, 5]. This has led to a similar increase in the number of people treated for schistosomiasis, with more than 66 million people treated for schistosomiasis in 2015, the vast majority in sub-Saharan Africa. However, even with increased donations, far fewer treatments are available than the number of people who are in need [5]. Thus, judicious distribution is warranted both in terms of the drug supply and the other costs associated with the logistics to deliver an MDA program.

In response to these considerations, the Schistosomiasis Consortium for Operational Research and Evaluation (SCORE) was formed in 2008 to address the needs of schistosomiasis control programs [6]. The emphasis of several SCORE projects was to provide an evidence base for program decisions related to MDA with praziquantel for *Schistosoma mansoni* and *Schistosoma haematobium* in areas with different levels of initial infection prevalence. This paper describes the results of different frequencies and timing of school-based MDA in villages in western Kenya with moderate (10% to 24%) initial prevalence of *S*. *mansoni* infection. Because WHO guidelines for interventions are based on community level infections in SAC [1, 2], we used a cluster randomized trial design to evaluate the impact of the different interventions on school-level prevalence and intensity of infection.

## Methods

### Study population

The study was conducted in Nyanza Province in 7 districts bordering Lake Victoria. This area was chosen based upon previous studies demonstrating schistosomiasis prevalence in the moderate range and in consultation with the Provincial Public Health Officer and with approval from the Ministry of Health. The area is semi-arid and bisected by the equator. Temperatures are reasonably consistent throughout the year. Over 96% of the population in these districts belongs to the Luo ethnic group.

We have previously demonstrated an inverse relationship between community distance from Lake Victoria and *S*. *mansoni* infection prevalence, suggesting that most schistosomiasis transmission in the area occurs through contact with the lake waters [7, 8]. No prior MDA with praziquantel had been attempted in any of the schools or communities in the study.

### Ethics statement

The study was approved by the Scientific Steering and Ethical Review Committees (ERC) of the Kenya Medical Research Institute (KEMRI); the Centers for Disease Control and Prevention institutional review board (IRB) relied on the KEMRI review. The IRB of the University of Georgia also administratively reviewed and approved the study. Written permission was obtained from the parents or guardians of children participating in the study. Informed assent was obtained from study participants less than 18 years of age. The trial is number 14849830 registered at ISRCTN.

### Study design

Protocols for SCORE randomized cluster studies to evaluate different MDA approaches including this project were developed during protocol harmonization meetings as previously described in detail [6]. In brief, an eligibility survey was conducted among 13–14 year old school children to identify villages with 10–24% initial prevalence of *S*. *mansoni* infection. One stool sample was collected from each of the 50 children participating per school, from which two Kato-Katz thick smears were prepared and examined microscopically to estimate infection prevalence.

**Fig 1:**
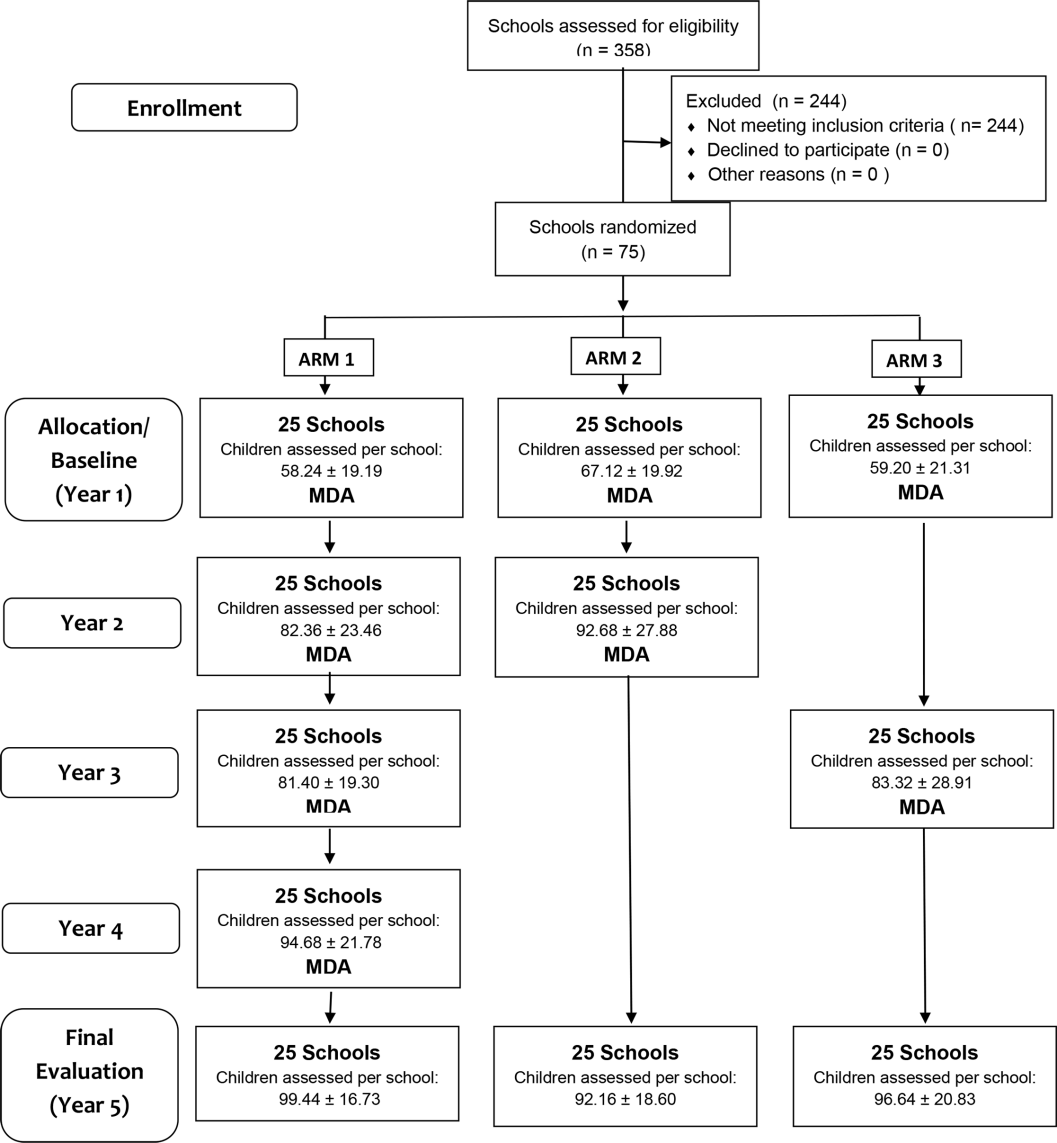
Flow diagram of trial design and number of schools and participants (mean ± standard deviation) in each school (cluster).

The number of schools per intervention arm and the number of children per school selected were based on an expected prevalence difference of 9.4% at the conclusion of the study [6]. Based on this power calculation, 75 primary schools in the appropriate prevalence range were identified by the study investigators and randomized into 3 study arms with 25 schools each using a simple random allocation program in SAS (Fig 1) [6]. For all study arms, baseline infection prevalence was determined for 9–12 year old children by sampling a target of 100 children per school. If more than 100 children in this age range were enrolled in a school, 100 children were randomly selected from the total eligible enrollment list. Three consecutive stool samples were sought from each participant, two Kato-Katz slides were prepared from each stool, and the number of schistosome eggs was counted on each slide. To compare effects of MDA on force of transmission, a single stool for preparation of two Kato Katz slides was also sought from up to 100 students in their first year of school. These children were typically between 5 and 8 years old.

Following baseline assessment of 9–12 year old children and first year students, praziquantel MDA (40mg/kg) was provided to all children in all 75 primary schools and administered by school health teachers who had been trained to use the praziquantel dose pole and recognize adverse responses to treatment. In the second year of the study, 9–12 year old children in schools in Arms 1 and 2 were assessed as at baseline (target of three stools, two slides per stool, for 100 children) and provided MDA. In year 3, 9–12 year old children in schools in Arms 1 and 3 were assessed and provided MDA. In year 4, only children in schools in Arm 1 were assessed and provided MDA. In year 5, 9–12 year old children in all schools were assessed in a final evaluation. In addition at year 5, 100 first year students per school were sought to provide a single stool for two Kato-Katz slides. Thus, children in schools in Arm 1 were assessed and provided MDA for each of 4 years with a final evaluation at year 5; children in schools in Arm 2 were assessed and provided MDA for the first 2 years and evaluated at year 5; and children in Arm 3 were assessed and provided MDA in years 1 and 3 with the final evaluation in year 5 (Fig 1).

Treatment coverage was estimated for each school in each year by comparing the number of children treated to the number of children enrolled in school, with a minimum coverage target of 90%. Primary school education in Kenya is free and the government has decreed it unlawful for children of the appropriate age to not be enrolled in school. Therefore, we considered the school enrollment records to be an accurate representation of the number of SAC in the community. The school health teacher administered the praziquantel and the individual class teacher recorded which students had been treated so that an accurate accounting of coverage was maintained.

### Data management and statistical analysis

Latitude and longitude coordinates were obtained at each school using Global Positioning System on Android phones and projected to UTM zone 36S for maps. Map was created with ArcGIS (version 10.3, ESRI, Inc., Redlands, CA). Parental permission and participant assent were obtained each sampling year. Study participants were assigned a unique identification number that was used to track all data using barcodes. Age, sex and village data were entered for participants using their unique identifier on Android phones. Stool cups with barcode labels were provided to the children in the morning and were returned before lunch. While stools on 3 consecutive days were sought from each 9–12 year old participant at each year they were assessed, it was not possible to get 3 stool samples from every child. Thus, infection intensity was determined based on the number of Kato-Katz slides (up to 6) available for each child. Android phones were used to read the barcode and the egg data were entered using EpiCollect [9]. If a child had at least 1 egg on any of the slides, he or she was considered positive for infection. Because the Kato-Katz method analyzes 41 mg of stool per slide, raw egg counts were multiplied by 24 to estimate the eggs per gram (epg) of feces. Infected children with an average epg <100 were considered to have light infections, 100–399 epg had moderate infections, and those with ≥400 epg had heavy infections according to WHO guidelines [1, 2]. Egg counts were truncated at 1008 epg.

Mean prevalence and intensity summary statistics by study arm and year accounted for village-level clustering via Taylor series linearization [10]. Changes over time and comparisons between arms were performed with negative binomial regression with generalized estimating equations (GEE) [11]. Communities at each year were the clustering factor and an exchangeable correlation structure was used. Adjusted models controlled for age and sex and were weighted by the inverse of the village sample size. Modeling results are reported as a prevalence ratio (PR) or arithmetic mean ratio (AMR). Because baseline intensity levels varied slightly, our main analyses assessed the changes over time. Thus, analyses of the changes over time compared the ratios of differences between the start and end of study in prevalence or intensity for each arm. Interclass correlation coefficients and design effects were calculated using mixed models [12, 13]. All analyses used the 5% level of significance.

## Results

**Fig 2:**
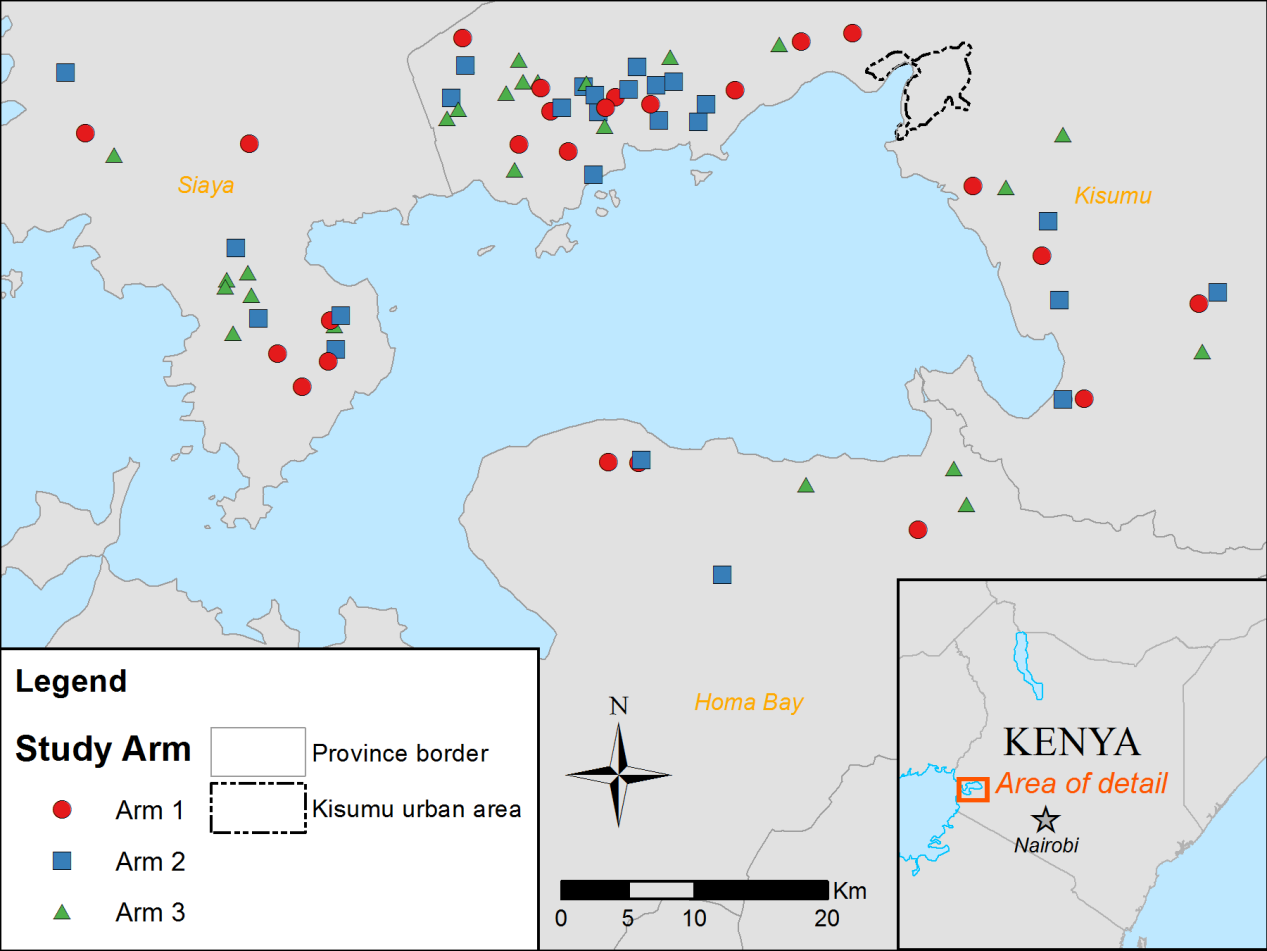
Location of schools included in study by arm, Western Kenya (n = 25 per arm). Map was created with ArcGIS (version 10.3, ESRI, Inc., Redlands, CA) using layers from ESRI data and Maps 10.1.

**Fig 3:**
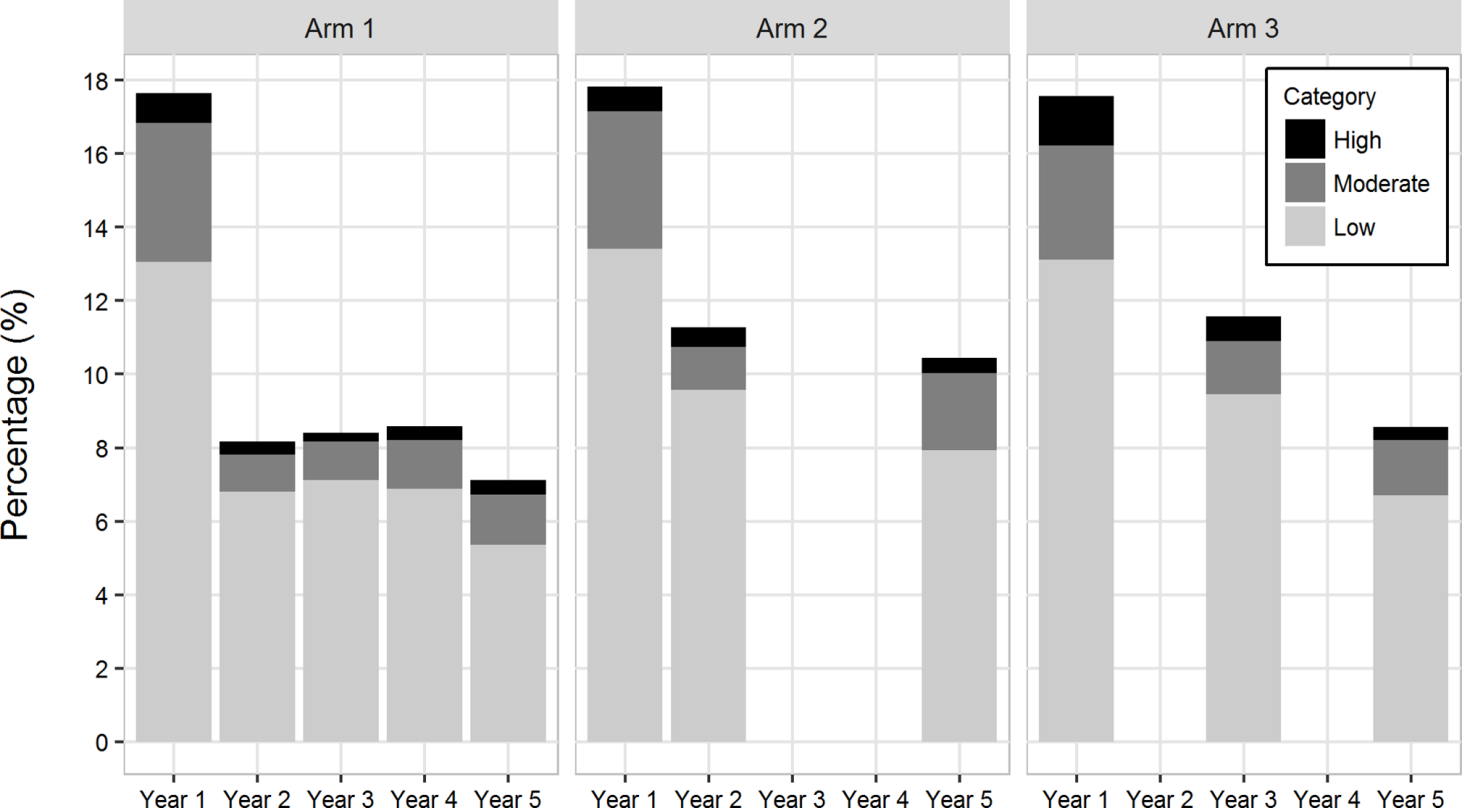
Overall mean prevalence and mean prevalence by infection intensity category by arm and by year. Total bar height represents mean *S*. *mansoni* infection prevalence in each year. Light grey represents the prevalence of individuals with low intensity infections (1–99 epg); darker grey represents prevalence of moderate intensity infections (100–399 epg); and black represents prevalence of individuals with high intensity infections (≥ 400 epg).

Fig 2 is a map of the 75 schools included in the study and shows the distribution of schools included in each of the study arms. The eligibility study was performed in 2010, with randomization of schools to arms and initiation of MDA in 2011; the study was completed in 2015. At baseline (year 1), the overall mean prevalence and infection intensity distributions (Fig 3) as well as the mean intensities of infection (Table 1) were similar among the three arms, suggesting that the randomization was effective. Between year 1 and year 5 (Table 2), there was a significant reduction in prevalence for all three arms (p ≤ 0.003) and intensity for arms 1 (p = 0.004) and 3 (p < 0.001), but not for arm 2 (p = 0.11). Interestingly, the largest drop for both prevalence and intensity in arm 1 was between years 1 and 2, corresponding to the first treatment, with subsequent years showing either no or relatively marginal further decreases (Fig 3, S1 Table). By contrast, the reductions in prevalence and intensity for arms 2 and 3 were more progressive. There were no statistically significant differences in the prevalence between arms at year 5 and the only significant difference in infection intensity was between arms 2 and 3, both of which had received 2 rounds of MDA (Table 3). The relative changes from baseline to year 5 were also not statistically different between arms (S2 Table), again suggesting that the different MDA regimens had a similar impact. Throughout the study, we were unable to achieve our target enrollment of 2500 per arm for any arm in any year. At baseline, enrollment per arm ranged between 1456 and 1678. Participation improved each year (S1 Table), with 2304 to 2486 children per arm enrolled in the final evaluation (Table 1). The intracluster correlation coefficient (95% CI) was 0.13 (0.09, 0.16) for prevalence and 0.01 (0.00, 0.02) for intensity. We observed a higher than expected design effect for the prevalence analysis (10.89, 95% CI 8.18–13.59). The design effect was not as pronounced for the analyses of infection intensity (2.07, 1.48–2.66). Since the assumed design effect in the power analyses was 2, the trial was severely underpowered to find differences in prevalence but was adequate for finding differences in infection intensity.

**Table 1:** Prevalence and intensity of infection in 9–12 year olds in each arm at baseline (Year 1) and final evaluation (Year 5).

		Year 1		Year 5	
Variable	Arm	n/N or N	% (CI) or mean (CI)	n/N or N	% (CI) or mean (CI)
Mean prevalence	Arm 1	257/1456	17.65 (13.01–22.29)	177/2486	7.12 (4.81–9.43)
(% infected, CI)	Arm 2	299/1678	17.82 (14.75–20.89)	241/2304	10.46 (7.58–13.35)
	Arm 3	260/1480	17.57 (11.76–23.37)	207/2416	8.57 (6.40–10.73)
Intensity	Arm 1	1456	16.08 (11.85–20.31)	2486	7.89 (3.78–12.01)
(mean epg, CI)	Arm 2	1678	14.45 (9.73–19.18)	2304	10.41 (6.36–14.46)
	Arm 3	1480	17.13 (11.11–23.15)	2416	7.89 (4.77–11.00)

n, Number of persons infected; N, Number of persons tested; CI, 95% confidence interval; epg, eggs per gram stool.

**Table 2:** Comparisons of change in 9–12 year old prevalence and intensity of *S*. *mansoni* infection between year 1 and year 5 for each arm, 25 villages per arm. Adjustment of PRs and AMRs are for age, sex, and village sample size.

	Prevalence			
Arm	Crude PR (CI)	p	Adjusted PR (CI)	P
1	0.41 (0.27, 0.61)	< 0.001	0.42 (0.27, 0.64)	< 0.001
2	0.56 (0.39, 0.81)	0.002	0.51 (0.33, 0.79)	0.003
3	0.48 (0.32, 0.71)	< 0.001	0.47 (0.32, 0.70)	< 0.001
	Intensity			
Arm	Crude AMR (CI)	p	Adjusted AMR (CI)	P
1	0.48 (0.27, 0.85)	0.01	0.41 (0.23, 0.75)	0.004
2	0.71 (0.42, 1.20)	0.20	0.52 (0.24, 1.16)	0.11
3	0.45 (0.27, 0.75)	0.002	0.39 (0.23, 0.66)	< 0.001

PR, Prevalence ratio; CI, 95% confidence interval; AMR, Arithmetic mean ratio.

**Table 3:** Comparisons of 9–12 year old prevalence and intensity of *S*. *mansoni* infection between arms at year 5. Adjustment of PRs and AMRs are for age, sex, and village sample size.

	Prevalence			
Comparison	Crude PR (CI)	P	Adjusted^c^ PR (CI)	p
Arm 2 vs. Arm 1	1.48 (0.96, 2.27)	0.08	1.49 (0.92, 2.39)	0.10
Arm 3 vs. Arm 1	1.17 (0.78, 1.74)	0.45	1.10 (0.72, 1.67)	0.66
Arm 3 vs. Arm 2	0.79 (0.54, 1.16)	0.23	0.74 (0.48, 1.13)	0.17
	Intensity			
Comparison	Crude AMR^d^ (CI)	P	Adjusted AMR (CI)	p
Arm 2 vs. Arm 1	1.35 (0.71, 2.57)	0.36	1.62 (0.83, 3.15)	0.16
Arm 3 vs. Arm 1	0.99 (0.52, 1.88)	0.98	0.86 (0.47, 1.60)	0.64
Arm 3 vs. Arm 2	0.73 (0.42, 1.27)	0.27	0.53 (0.29, 0.98)	0.04

PR, Prevalence ratio; CI, 95% confidence interval; AMR, Arithmetic mean ratio.

Treatment coverage was monitored to ensure that relative effects of MDA regimens were not influenced by differences in the proportion of individuals receiving treatment. Mean coverage did not differ by arm or by year for schools scheduled to receive MDA that year (Table 4). While some schools in each arm did not achieve the target of 90% of students receiving treatment in a given year, there were only two schools (one in arm 1 and one in arm 3) that did not have at least 90% coverage in more than one treatment year.

**Table 4:** Mean treatment coverage percentage, range, and number (n) of schools with less than 90% coverage in each arm in each year.

	Year 1	Year 2	Year 3	Year 4
Arm 1	92.7, 54.5–100 (4)	96.2, 75.0–132 (2)	93.8, 77.2–100 (3)	93.6, 82.3–100 (4)
Arm 2	92.1, 68.0–100 (5)	97.6, 90.9–106 (0)	ND	ND
Arm 3	93.1, 63.2–100 (3)	ND	92.4, 75.0–99.7 (6)	ND

ND, not done.

Prevalence and intensity of infection were also measured in first year students at baseline and year 5 (Table 5). Because pre-school children were not included in the school-based MDA, any differences in first year student infection levels might suggest changes in environmental exposure or force of transmission. There were no statistically significant differences between arms at the two time points first year student infections were measured. There were also no differences between years 1 and 5 within a given arm, suggesting that children had similar risk for infection at both time points.

**Table 5:** Prevalence and intensity of *S*. mansoni infection for first year students in each arm at baseline (Year 1) and final evaluation (Year 5).

		Year 1		Year 5	
Variable	Arm	n^a^/N^b^ or N	% (CI^c^) or mean (CI)	n/N or N	% (CI) or mean (CI)
Mean prevalence	Arm 1	21/474	4.43 (2.34–6.52)	38/732	5.19 (3.42–6.96)
(% infected, CI)	Arm 2	31/574	5.40 (3.62–7.18)	60/664	4.52 (2.43–6.61)
	Arm 3	41/561	7.31 (5.29–9.33)	26/671	3.87 (2.26–5.48)
Intensity	Arm 1	474	10.81 (2.22–19.40)	732	8.81 (3.82–13.80)
(mean epg, CI)	Arm 2	574	7.23 (2.71–11.76)	664	9.99 (0.00–21.44)
	Arm 3	561	7.51 (4.41–10.61)	671	8.47 (2.89–14.06)

n, Number of persons infected; N, Number of persons tested; CI, 95% confidence interval; epg, eggs per gram stool.

## Discussion

In a cluster randomized trial, we evaluated different frequencies and timing of school-based MDA on infection levels among 9–12 year old children living in communities with moderate initial prevalence *S*. *mansoni* infections. We found significant decreases in prevalence of infection over time within all arms and significant decreases in intensity of infection within the arms that received treatment every year or every other year. However, four treatments did not result in significantly lower prevalence or intensities of infection compared to treatment every other year over a 5 year period. These data suggest that biennial treatment may have similar benefits as annual treatment in schistosomiasis control programs in moderate prevalence areas, making it possible to provide MDA to twice as many schools with the same resources as using annual MDA. However, we observed that for all arms it was only possible to reduce prevalence to approximately half of initial prevalence, regardless of whether two or four rounds of MDA were provided. This is consistent with the proposition that MDA alone, while able to sustain morbidity control, will not be sufficient to effect schistosomiasis elimination and will need supplementation with other control measures such as improved water and sanitation systems, snail control, or immunization if an effective vaccine one day becomes available [14]. However, it was possible to transition communities in arms 1 and 3 from the WHO-defined “moderate-risk” category to “low risk” (average infection levels < 10% prevalence) using school based MDA alone although there was a small number of children in some villages that still had high intensity infections at year 5.

In the final evaluation in year 5, first year students, who were not in school in any previous years and therefore would never have received MDA, had similar prevalence and infection intensity levels as the first year students at the study baseline. This result suggests that at this level of infection in the community, the school based MDA did not influence the force of transmission of schistosomiasis to which these children were exposed.

The main limitation of this study was a failure to achieve the target sample size for monitoring infections in either 9–12 year olds or first year students. A total of 2500 students was targeted but never reached in any arm in any year. Sampling in the final evaluation came the closest (2304 to 2486 students per arm) but none of the arms in the baseline sampling year enrolled more than 1700 children. The sample size we achieved for first year students was even worse with only 474 to 732 children tested for any of the first year student the time points. This failure to achieve the targeted sample sizes was primarily a function of lower than anticipated SAC populations rather than refusal of any subgroup to participate or children not attending school in these communities. However, because the sampling unit was the school, which remained 25 for all time points and arms, we do not believe that the lower than anticipated number of participants would have reduced the probability of detecting a difference when a true difference existed, although it could have decreased the precision of the results and contributed to the unanticipatedly large design effect that we observed. Despite not achieving our targeted sample sizes, this study is the largest randomized trial comparing schistosomiasis MDA regimens to date.

Another possible limitation is that while these studies were being performed, a parallel study of school-based and community-wide MDA was being conducted in communities with higher initial prevalence (>25% infection among school children) in the same districts (manuscript in preparation). Most of the communities in the other study were closer to Lake Victoria as there is a direct relationship between prevalence and proximity to the lake [7, 8]. Thus, it is possible that MDA in communities between the lake and the schools described in this study could have affected how students in the schools described here were exposed. However, the similarity of infection levels in first year students at baseline and final evaluation argues against the parallel study confounding these results.

In conclusion, school-based MDA with praziquantel was effective at reducing schistosomiasis prevalence in 9–12 year old Kenyan school children from “moderate-risk” to “low-risk” according to current WHO guidelines designed to prevent severe morbidity [1, 2]. Biennial treatment appeared as effective as annual treatment to accomplishing this reduction while 2 rounds of treatment followed by 2 years without treatment was slightly less effective. However, the data also suggest that MDA alone is unlikely to allow countries to achieve the elimination goals set forth by WHA resolution 65.21. Nevertheless, we anticipate that the results from this and the other SCORE MDA studies for gaining and sustaining control will contribute to the evidence base for development of new guidelines that will be useful for schistosomiasis control and elimination and assist NTD program managers to allocate resources according to their goals.

## Supporting information

S1 Table Prevalence and intensity of *S. mansoni* infection for 9–12 year olds in each arm, years 2–4. (DOCX)

S2 Table Comparison of changes in *S. mansoni* infection from year 1 to year 5 between arms, prevalence and intensity, 9–12 year olds. Adjustment of PRs and AMRs are for age, sex, and village sample size.
(DOCX)

S3 Table CONSORT 2010 checklist of information to include when reporting a cluster randomized trial. (DOCX)
