# Supplementary material for: Cluster randomized trial comparing school-based mass drug administration schedules in areas of western Kenya with moderate initial prevalence of Schistosoma mansoni infections
Source: PLoS Negl Trop Dis. 2017 Oct 23;11(10):e0006033. doi: 10.1371/journal.pntd.0006033 (PMC5667887; doi:10.1371/journal.pntd.0006033)
Supplement: S1 Table — (DOCX) [file pntd.0006033.s001.docx]

Supplemental Table 1. Prevalence and intensity of *S. mansoni* infection for 9-12 year olds in each arm, years 2-4.

|  |  | Year 2 | | Year 3 | | Year 4 | |
| --- | --- | --- | --- | --- | --- | --- | --- |
| Variable | Arm | n/N or N | % (CI) or mean (CI) | n/N or N | % (CI) or mean (CI) | n/N or N | % (CI) or mean (CI) |
| Mean prevalence | Arm 1 | 168/2059 | 8.16 (6.01 -10.31) | 171/2035 | 8.40 (6.37 – 10.44) | 203/2367 | 8.58 (4.74 - 12.41) |
| (% infected, CI) | Arm 2 | 261/2317 | 11.26 (7.71 – 14.82) |  |  |  |  |
|  | Arm 3 |  |  | 241/2083 | 11.57 (8.45 - 14.68) |  |  |
| Intensity | Arm 1 | 2059 | 5.55 (2.13 – 8.97) | 2035 | 5.21 (2.83 - 7.58) | 2367 | 6.58 (3.79 – 9.36) |
| (mean epg, CI) | Arm 2 | 2317 | 7.71 (3.69 – 11.72) |  |  |  |  |
|  | Arm 3 |  |  | 2083 | 9.46 (5.81 - 13.12) |  |  |

n, Number of persons infected; N, Number of persons tested; CI, 95% confidence interval; epg, eggs per gram stool.
