# Supplementary material for: Cluster randomized trial comparing school-based mass drug administration schedules in areas of western Kenya with moderate initial prevalence of Schistosoma mansoni infections
Source: PLoS Negl Trop Dis. 2017 Oct 23;11(10):e0006033. doi: 10.1371/journal.pntd.0006033 (PMC5667887; doi:10.1371/journal.pntd.0006033)
Supplement: S2 Table — Adjustment of PRs and AMRs are for age, sex, and village sample size. (DOCX) [file pntd.0006033.s002.docx]

Supplemental Table 2. Comparison of changes in *S. mansoni* infection from year 1 to year 5 between arms, prevalence and intensity, 9-12 year olds. Adjustment of PRs and AMRs are for age, sex, and village sample size.

|  | Prevalence | | | |
| --- | --- | --- | --- | --- |
| Comparison | Crude PR^a^ (CI^b^) | p | Adjusted PR (CI) | p |
| Arm 2 vs. Arm 1 | 1.38 (0.81, 2.36) | 0.24 | 1.23 (0.66, 2.26) | 0.51 |
| Arm 3 vs. Arm 1 | 1.18 (0.67, 2.06) | 0.56 | 1.14 (0.64, 2.03) | 0.65 |
| Arm 3 vs. Arm 2 | 0.85 (0.50, 1.46) | 0.57 | 0.93 (0.51, 1.69) | 0.82 |
|  | Intensity | | | |
| Comparison | Crude AMR^c^ (CI) | p | Adjusted AMR (CI) | p |
| Arm 2 vs. Arm 1 | 1.47 (0.68, 3.22) | 0.33 | 1.26 (0.47, 3.41) | 0.64 |
| Arm 3 vs. Arm 1 | 0.94 (0.44, 2.03) | 0.87 | 0.93 (0.42, 2.06) | 0.86 |
| Arm 3 vs. Arm 2 | 0.64 (0.30, 1.34) | 0.23 | 0.74 (0.29, 1.89) | 0.52 |

PR, Prevalence ratio; CI, 95% confidence interval; AMR, Arithmetic mean ratio
